# Correction: Choi et al. Feasibility and Effect of Electroacupuncture on Cognitive Function Domains in Patients with Mild Cognitive Impairment: A Pilot Exploratory Randomized Controlled Trial. *Brain Sci*. 2021, *11*, 756

**DOI:** 10.3390/brainsci11101334

**Published:** 2021-10-11

**Authors:** Yujin Choi, In-Chul Jung, Ae-Ran Kim, Hyo-Ju Park, Ojin Kwon, Jun-Hwan Lee, Joo-Hee Kim

**Affiliations:** 1 Clinical Medicine Division, Korea Institute of Oriental Medicine, Daejeon 34054, Korea; choiyujin@kiom.re.kr (Y.C.); arkim@kiom.re.kr (A.-R.K.); mable@kiom.re.kr (H.-J.P.); cheda1334@kiom.re.kr (O.K.); omdjun@kiom.re.kr (J.-H.L.); 2 Department of Neuropsychiatry, College of Korean Medicine, Daejeon University, Daejeon 34520, Korea; npjeong@dju.kr; 3 Korean Medicine Life Science, University of Science & Technology (UST), Campus of Korean Institute of Oriental Medicine, Daejeon 34054, Korea; 4 Department of Acupuncture and Moxibustion Medicine, College of Korean Medicine, Sangji University, Wonju-si 26339, Korea; 5 Research Institute of Korean Medicine, Sangji University, Wonju-si 26339, Korea

The authors wish to make the following correction to [1]. The heading of Table 5 of the manuscript was misplaced, and two adverse events which are unlikely related to the intervention in the EA group were omitted. The corrected table is as follows.

The authors would like to apologize for any inconveniences caused to the readers by these changes.

## Figures and Tables

**Table 5:** Adverse events (all causalities) reported during the study.

	EA Group (*n* = 20)	Sham EA Group (*n* = 20)	Usual Care Group (*n* = 20)
**Adverse events (possibly or probably related)**			
Headache	1	1	1
Bruise	4	0	0
Pruritus	2	0	0
**Severity of AEs**			
Mild	17	8	1
Moderate	2	0	0
Severe	1	1	0
**Causality of AEs**			
Definitely related	0	0	0
Probably related	6	0	0
Possibly related	1	1	1
Unlikely related	9	1	0
Definitely not related	4	7	0
Total number of participants with AE	10	5	1
Total number of AEs	20	9	1
Total number of AEs (intervention-related)	7	1	1
Total number of SAEs	1	1	0
Total number of SAEs (intervention-related)	0	0	0
Total number of interventions (or visits)	407	371	88
